# Economic consequences of migraine in Sweden and implications for the cost-effectiveness of onabotulinumtoxinA (Botox) for chronic migraine in Sweden and Norway

**DOI:** 10.1186/s10194-020-01162-x

**Published:** 2020-08-12

**Authors:** Amanda Hansson-Hedblom, Isabelle Axelsson, Lena Jacobson, Joakim Tedroff, Fredrik Borgström

**Affiliations:** 1 Quantify Research AB, Stockholm, Sweden; 2 Allergan Norden AB, Stockholm, Sweden; 3 Neuroenheten Utsikten, Stockholm, Sweden

**Keywords:** Migraine, Headache, onabotulinumtoxinA, Botox, Cost-effectiveness, Cost of illness, Burden

## Abstract

**Background:**

Migraine is a common and incapacitating condition, with severe impact on the quality of life (QoL) of the afflicted and their families, and negative economic consequences through decreased workforce participation, reduced functional ability and elevated healthcare costs. This study aimed to describe the economic consequences of migraine in Sweden using cost of illness survey data and, based on this data, assess the cost-effectiveness of onabotulinumtoxinA (Botox) for the treatment of chronic migraine in Sweden and Norway.

**Methods:**

A survey study was conducted in Swedish migraine patients, with questions on patient characteristics, headache frequency and severity, effect on daily activities and work, QoL, health resource utilization, and medication use. Resulting costs were estimated as annual averages over subgroups of average monthly headache days. Some results were used to inform a Markov cost-effectiveness chronic migraine model. The model was adapted to Sweden and Norway using local data. The analysis perspective was semi-societal. Results’ robustness was tested using one-way, structural, and probabilistic sensitivity analyses.

**Results:**

Results from the cost of illness analysis (*n* = 454) indicated a clear correlation between decreased QoL and increased costs with increasing monthly headache days. Total annual costs ranged from EUR 6221 in patients with 0–4 headache days per month, to EUR 57,832 in patients with 25–31. Indirect costs made up the majority of costs, ranging from 82% of total costs in the 0–4 headache days group, to 91% in 25–31 headache days. The cost-effectiveness analyses indicated that in Sweden, Botox was associated with 0.223 additional QALYs at an additional cost of EUR 4126 compared to placebo, resulting in an incremental cost-effectiveness ratio (ICER) of EUR 18,506. In Norway, Botox was associated with 0.216 additional QALYs at an additional cost of EUR 4301 compared to placebo, resulting in an ICER of EUR 19,954.

**Conclusions:**

In people with migraine, an increase in monthly headache days is clearly related to lower QoL and higher costs, indicating considerable potential costs-savings in reducing the number of headache days. The main cost driver for migraine is indirect costs. Botox reduces headache days and is a cost-effective treatment for chronic migraine in Sweden and Norway.

## Background

Migraine is a chronic, neurologic headache disorder characterised by recurrent attacks of varying intensity, duration and symptoms. In addition to headache and pain, symptoms frequently include deterioration of physical functions, nausea, vomiting, and sensitivity to light and sounds. A migraine attack typically lasts from 4 to 72 h if left untreated [1]. In the adult Swedish population 17% of women and 10% of men suffer from migraine [2]. Similarly in Norway, the corresponding is approximately 15% of women and 7% of men [3].

Migraine is an incapacitating disease with a substantial quality of life (QoL) loss and economic burden. The latter partially through increased healthcare costs, but mainly due to lost productivity and reduced functioning. Migraine-related productivity loss consists of both absenteeism and decreased productivity while on the job (presenteeism). Previous studies have shown a clear relationship between the number of headache days and increased costs and decreased QoL [4–10]. For example, a recent Italian study showed that the direct annual cost of CM patients is 4.8 times higher than the cost of EM patients [11]. Despite the high prevalence and the apparent and substantial burden, few current health economic studies have explored the real-world impact of migraine and headaches in the Nordics. The Eurolight study – conducted over a decade ago – estimated the cost of headaches in the European Union (EU) to EUR 173 billion annually. Migraine accounted for 64% of the cost, with indirect costs comprising 90% of the total [12]. A Swedish survey study found that increased monthly migraine days are associated with significant QoL losses and cost increases, with productivity losses comprising the vast majority (80%) of the total [13]. The study identified potential for significant QoL improvement and cost decreases related to the reduction of migraine days.

Pharmaceutical treatments for migraine in Sweden and Norway include acute medications for symptom relief during migraine attacks and prophylactic medications intended to prevent and reduce severity of future attacks. Acute medications include analgesics, as well as triptans, antiemetics and ergotamine [1, 14, 15].

Prophylactic treatment is considered for patients with frequent or severe migraine attacks, despite acute treatment. For first choice of prophylactic treatment, beta-blockers are recommended, while other alternatives include antiepileptics, antidepressants, angiotensin 2 blockers or ACE inhibitors, calcium antagonists, angiotensin-II receptor inhibitors or OnabotulinumtoxinA (Botox).

Botox is indicated in both Sweden and Norway for symptom relief in adults fulfilling criteria for chronic migraine (CM) in patients who have responded inadequately or are intolerant of prophylactic migraine medications [16]. CM is defined as > 3 months of headaches occurring on ≥15 days per month, of which ≥8 days are with migraine [17]. In Sweden, Botox is reimbursed according to its approved migraine indication. In Norway, Botox is reimbursed for CM in patients who previously tried a beta-blocker (propranolol, metoprolol or atenolol) and topiramate.

Calcitonin gene-related peptide (CGRP) inhibitors erenumab (Aimovig), galcanezumab (Emgality) and fremanezumab (Ajovy) are indicated for prevention of migraine in adults with at least four migraine days per month in both Sweden and Norway. In Sweden, erenumab and fremanezumab are reimbursed in patients with CM who have responded insufficiently or are intolerant to at least two prophylactic migraine treatments. Galcanezumab is not available in Sweden. In Norway, erenumab, fremanezumab and galcanezumab are reimbursed for CM in patients who have responded insufficiently or are intolerant to at least three different classes of prophylactic migraine treatments.

Several clinical and real-world studies have shown that treatment with Botox reduces the number of headache days in patients with CM as well as days absent from work and school [18–21]. The safety and efficacy of Botox in CM in adult patients was evaluated in randomized, double-blind, placebo-controlled clinical trials PREEMPT 1 and 2: Phase 3 REsearch Evaluating Migraine Prophylaxis Therapy (PREEMPT) – the largest clinical program investigating the use of Botox as prophylactic treatment for CM [18]. International, multicentre, open-label long-term prospective study COMPEL (The Chronic Migraine OnabotulinumtoxinA Prolonged Efficacy open Label) provided additional clinical evidence of efficacy and long-term safety and tolerability [22]. European, open-label, multicentre, prospective, noninterventional study REPOSE provided evidence of a sustained reduction in headache-day frequency and significant improvements in QoL in CM patients treated with Botox [23].

While previous cost-effectiveness (CE) analyses of Botox in chronic migraine have been conducted in the United Kingdom (UK) [24, 25] and Italy [26] there is a lack of Nordic CE studies. CE outcomes are highly dependent on country specific factors such as e.g. national health system, clinical practice and local cost and utility data. Therefore, previous European CE studies are inadequate for drawing conclusions on the Nordic situation. This study described the economic consequences of migraine in Sweden using cost of illness (COI) survey data. Additionally, results from the COI study were used to populate a CE analysis to assess the CE of Botox for the treatment of CM in Sweden and Norway.

## Methods

### Cost of illness survey

A survey was conducted May – June 2018 among Swedish individuals suffering from headaches and migraine. All members of the Swedish patient association Huvudvärksförbundet (“*the Headache Association*”) were invited to participate in an online survey by email, in collaboration with the association [27]. The purpose of establishing a sample population using a patient organisation was to reach patients with diagnosed migraine who are likely to provide reliable answers to survey questions. Huvudvärksförbundet encourages their members to use migraine diaries, further reducing the likelihood of recall bias. The most plausible option to obtain these types of data on a patient level while capturing the burden over different severity levels is to ask patients directly. Sourcing e.g. resource use from the literature would have likely only captured the most severe patients. However, an intrinsic issue with purposive sampling using patient organisations is the risk that the patient sample differs from the general migraine population. This risk was handled by consulting Swedish and Norwegian clinicians, who confirmed that the sample corresponded well to general migraine populations. The survey contained patient characteristics questions such as headache diagnosis, sex, and year of birth; headache frequency and severity; questions regarding the effect of headache on daily activities and work, quality of life related questions such as the EuroQol visual analogue scale (EQ-VAS), and questions regarding health resource utilization and medication use. Questions on headache frequency, severity and effect on daily activities and work were based on the Migraine Disability Assessment (MIDAS) questionnaire [28]. The survey was initiated with a screening question to ensure that all respondents suffered from some type of headache. Patients were selected for study inclusion on the basis they had a migraine diagnosis. Survey participants were grouped according to average number of headache days per month (0–4 days, 5–9 days, 10–14 days, 15–24 days and 25–31 days) and results were presented for each group. This categorisation enables analyses in subgroups of patients with episodic and chronic migraine, with evenly distributed categories of 5 days. The reason for using headache days rather than migraine days was threefold. Firstly, migraine often presents with both migraine days and headache days; with migraine being the disease and different types of headaches being the symptoms. Secondly, it is not always possible for patients to distinguish between headache and migraine days. And thirdly, the primary endpoint in the pivotal trials for Botox in patients with CM was the number of headache days per month.

Direct and indirect costs were estimated separately. Direct costs included costs of health resource use and the cost of preventive and acute pharmaceutical headache treatments. The survey requested the number of hospitalizations and number of planned or acute visits to a specialist clinic or a primary health care centre (“Vårdcentral”) in the last 3 months. The average number of visits per monthly headache days group were multiplied with the per-visit cost sourced from the Swedish Southern Health Care Region’s (“Södra sjukvårdsregionen”) pricelist, and then multiplied by four to obtain annual costs. Refer to Table 1 for the unit costs. Pharmaceutical treatment costs were estimated using price per defined daily dose (DDD) for each reported treatment. Patients on preventive treatment were assumed to use the treatment for 1 year. Acute treatments were assumed to be used for as many days as the respondent reported headache days.

**Table 1:** Unit costs (EUR 2019)

Type	Sweden		Norway	
	Cost (EUR)	Source	Cost (EUR)	Source
***Treatments***				
Botox (PSP)	347	[29]	335	[30]
Placebo	–	–	–	–
***Administration***				
Neurology consultant appointment	279	[31]	248	[32]
Specialist nurse appointment	86	[33]	50	[34]
***Resources***				
GP appointment	155	[31]	33	[35]
A&E visit	279	[31]	73	[35]
Hospitalisation (per day)	665	[31]	771	[32]
Cost of triptans (per attack)	2	[29]	4	[36]
***Productivity***				
Average hourly wage	40	[37, 38]	45	[34]
***Additional inputs used in COI analyses***				
Value of spare time/hour	11	[39]	–	–
Value of spare time, retail price/hour	22	[40, 41]	–	–
GP appointment (acute)	369	[31]	–	–

***Key***: *A&E* Accident and emergency; *GP* General practitioner; *PSP* Pharmacy selling price; *VAT* Value added tax

***Note***: In Norway the VAT for pharmaceuticals is 25%, while there is no VAT for prescription pharmaceuticals in Sweden. The analyses were undertaken excluding VAT. All Swedish unit costs except average wage are sourced from 2019. Average wage was inflated from 2018 to 2019 values using CPI from Statistics Sweden [42]. Some of the Norwegian unit costs have been inflated to 2019 values using CPI from Statistics Norway [43]. All costs are presented in EUR, using ECB average annual 2019 NOK/EUR and SEK/EUR exchange rates [44, 45]

Indirect costs were estimated based on MIDAS as the value of lost production due to absenteeism and presenteeism using the Swedish average hourly wage including employer contributions [37, 38]. Absenteeism – e.g. being absent from work or school – was costed using the daily average wage for each day the respondent was not present. Presenteeism was costed using 50% of the daily average wage for each respondent indicated day of working at reduced ability due to headache. This is a conservative approach, as the survey question related to presenteeism requested an indication of number of days present at work or school but performing at half or lower capacity due to headache. Foregone household work was costed using a Swedish estimate of the value of spare time (EUR 11.18 per hour) sourced from the Swedish Transport Agency (“*Transportstyrelsen*”) [39], inflated and converted to EUR 2019 using Consumer Price Index (CPI) from Statistics Sweden [42] and European Central Bank (ECB) 2019 average annual SEK/EUR exchange rate [44]. It was assumed that 4 h per day were spent on household work on days unaffected by migraine, based on a Statistics Sweden survey on time spent doing housework [46], weighted by the gender proportion in our sample. This approach was selected as it was preferable to use a Swedish estimate. It should however be noted that this was a conservative approach, given that higher cost estimates have been suggested in the literature [47], and considering alternative approaches. Such approaches include e.g. the market wage rate approach where the value of a foregone hour of household work is costed using the average market wage, and the retail market value approach using the cost of purchasing professional cleaning services. A sensitivity analysis using the latter approach was undertaken, costing foregone household work with an hourly cost of EUR 22, sourced from the mean cost of household work from two of the largest housekeeping companies in Sweden [40, 41].

### Cost-effectiveness analysis

#### Model description

The CE analyses were performed using a Markov model developed for analyses in the UK [24] which was adapted to a Nordic setting. The analysis compared Botox to placebo in Sweden and Norway. Placebo was used as a comparator in the absence of an established standard of care for CM and since placebo was the comparator in the PREEMPT trials [18–21]. This was a conservative approach, as placebo patients in the trials saw considerable reductions in number of headache days, which is unlikely to be observed in best supportive care in a real-world setting. The trial response was likely due to placebo patients being allowed to stay on their usual acute headache medications while receiving saline solution injections. In these analyses, placebo was assumed to consist of consultant appointments to fit rescue medication. A sensitivity analysis was undertaken, as far as possible mirroring previous Swedish and Norwegian CE analyses of erenumab and fremanezumab versus placebo [48, 49]. This was done to indirectly compare Botox with CGRP inhibitors in lack of sufficient comparative efficacy data for a direct comparison.

The analysis took a semi-societal perspective, including costs regardless to who they were incurred. In line with TLV recommendations [50, 51] indirect costs were only included in a sensitivity analysis. For the purpose of non-discrimination of patient populations far from and outside the labour market (e.g. the elderly), the TLV considers indirect costs with great caution.

**Fig. 1:**
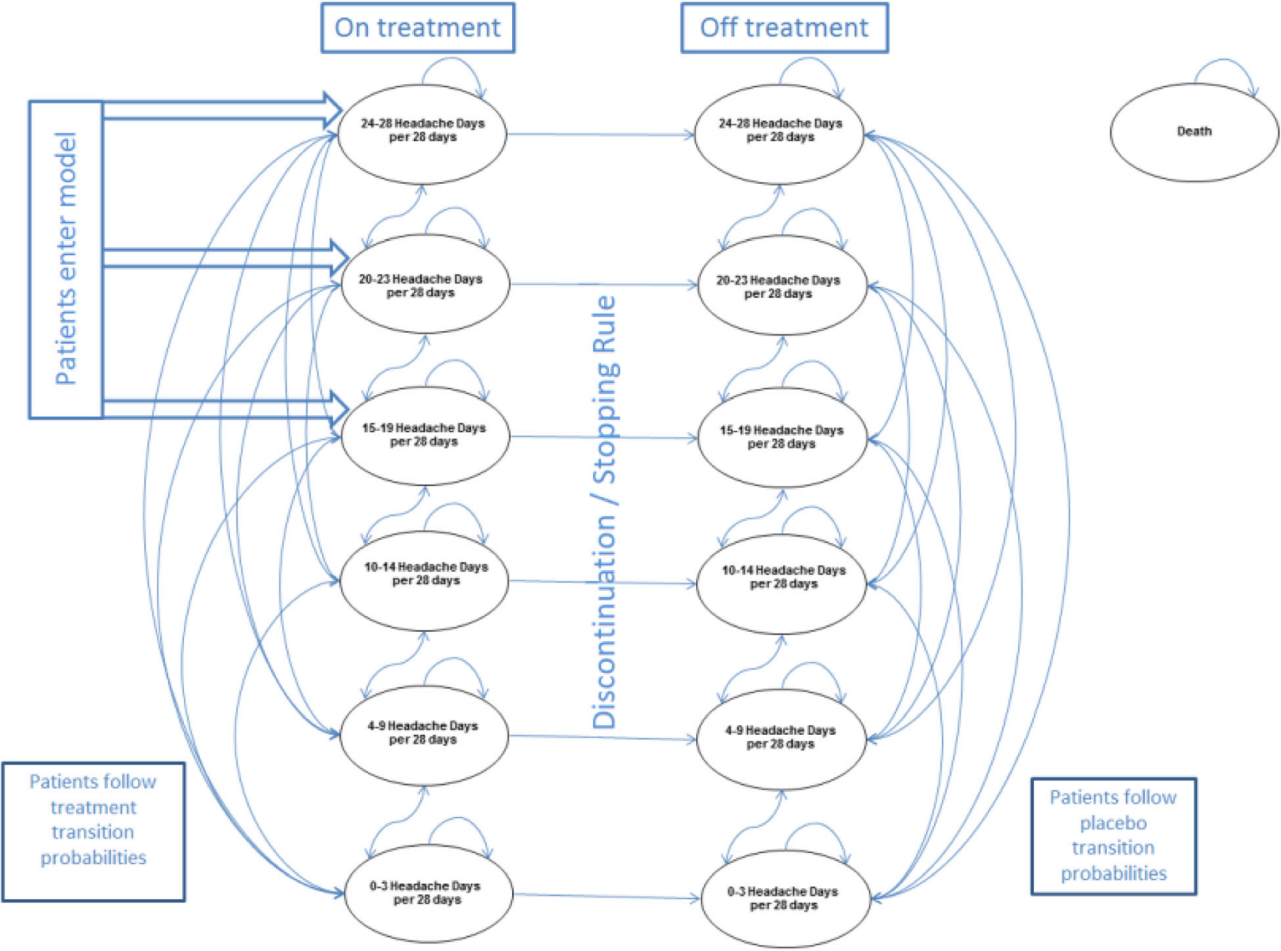
Model structure

The model cycle length was 12-weeks, in line with the frequency of Botox and placebo administrations in the clinical trials. At the beginning of each cycle, patients could transition between 13 health states (depicted in Fig. 1) and were then assumed to remain in the same health state for the duration of the cycle. The health states were split into six “on treatment” states and six “off treatment” states, stratified based on number of headache days per 28 days as follows: 0–3, 4–9, 10–14, 15–19, 20–23, and 24+ headache days. The three states with the lowest number of headache days correspond to episodic migraine (EM) and the three states with the highest number of headache days correspond to CM. At simulation initiation, the patients were distributed in the CM health states, with the proportion of patient in each state sourced from a post-hoc analysis of the clinical trials [18]. Death is an absorbing health state in the model. Figure 1 summarises the health states and all possible transitions. The base case analysis used a 10-year time horizon, in line with Swedish clinical expert input. This fairly long time horizon was selected as CM is a chronic disease and is in line with a previous CE analysis in CM, in which The Norwegian Medicines Agency (NOMA) assessed erenumab [49].

In absence of sufficient Botox response, a stopping rule was applied as it is unlikely that clinicians would continue to treat patients experiencing unsatisfactory results. This was confirmed by Nordic clinical experts. In the base case scenario, patients who did not see a ≥ 30% reduction in headache days within the first two cycles (24 weeks) were assumed to discontinue treatment and remain in the “no treatment” health states on placebo transition probabilities for the remainder of the simulation. The transition probabilities were calculated from the clinical trial database. In each cycle, patients may discontinue, either as a result of the stopping rule, or based on discontinuation probabilities estimated from the clinical trials. Discontinued patients incurred no Botox treatment costs. The model did not include any increased mortality linked to migraine as there is no support in the literature for migraine related mortality. General population mortality was sourced from Statistics Sweden and Statistics Norway life tables. With a few exceptions, transition probabilities were calculated as follows:

$$ probability\ of\ moving\ from\ state\ A\  to\ state\ B=\frac{number\ of\ patients\  who\  moved\ from\ state\ A\  to\ state\ B}{total\ number\ of\ patients\  who\  started\ in\ state\ A} $$

However, in the clinical trial, there were some cases of no patients starting in certain health states, rendering it impossible to calculate probabilities. In this case, it was assumed that all patients in the model who started a cycle in such state remained there.

In the trial, both arms saw a large decrease in headache days over the first 12 weeks which was not representative for later treatment periods. The initial dramatic drop weeks 0–12 was followed by a steady decline in the weeks 12–24. Therefore, the first two model cycles (24 weeks) were modelled using two separate sets of transition probabilities: weeks 0–12 and 12–24. The trial was unblinded at week 24 and placebo patients were switched to Botox. Therefore, transition probabilities for weeks 12–24 were repeated for placebo patients in the model cycles beyond 24 weeks. For Botox, open-label data was used to estimate transition probabilities beyond 24 weeks.

The model contains transition probabilities calculated from the clinical trial database for several different patient populations, defined by the number of previously tried oral prophylactics and including or excluding patients overusing acute medications. In the base case all patients were included. This assumption was made due to the relatively low patient numbers in PREEMPT rendering subgroup analyses less robust and to be in line with the original model analysis for the UK [24]. Sensitivity analyses were done to analyse the impact of assumptions (e.g. related to stopping rule, population and time horizon) and to mirror analyses for CGRP inhibitors referenced in Swedish and Norwegian reimbursement decision reports. In both Sweden and Norway, the mean age of the population was 42 years and 87.5% of the patients were female, in line with the clinical trials. Local clinicians have confirmed that this corresponds well to clinical practice.

#### Model inputs

Participants in the survey reported EQ-VAS which was used to estimate utilities per health state to inform the model. A total of 454 patients were selected for the analysis on the basis that they had diagnosed migraine and excluding apparent outliers (*n* = 10). Respondents were considered outliers if they reported more headache days than actual days per month, or reported implausibly high levels of healthcare utilisation. The mean VAS score was estimated for each health state, as reported in Table 5. This approach used the same utility values in both arms, which given the difference seen in patient reported outcomes (PROs) throughout clinical trials PREEMPT 1 and PREEMPT 2 is likely to be a conservative assumption.

Utility values mapped to EQ-5D from the Migraine Specific Questionnaire (MSQ) (based on [52]) included in the PREEMPT clinical trials [24] as well as EQ-5D scores from REPOSE using UK value sets [24] and from the International Burden of Migraine Study (IBMS) [53] were explored in scenario analyses.

A minimum of 155 Allergan Units of Botox were assumed administered in 31–39 injections in line with PREEMPT and the summary of product characteristics (SPC). Botox is available in three vial sizes, 50 units, 100 units and 200 units. It was assumed that one 200 Allergan Unit vial was used per dose, with no vial sharing possible, as confirmed by local clinicians.

Resource use included the number of primary care provider visits, neurologist/headache specialist visits, emergency department visits, hospital visits and hospitalisations per 3-month period. Except for hospitalisations and triptan usage which were informed by IBMS, all resource use was based on the COI survey (Table 5). EM was associated with 0.03 hospitalisations per 3-month period and CM to 0.09. The 0–3 headache days health state was associated with 1.88 triptans per 3-month period, the 4–9 and 10–14 health states to 5.07 triptans, and the CM health states to 7.29. Each type of resource use informed by the COI study was estimated as mean per health state in the 454 patients with diagnosed migraine. Both Botox and placebo treatment in the model was associated to neurologist visits (assumed twice per year in the Botox arm and once per year for placebo). Botox treatment was also associated with a 30 min specialist nurse visit every cycle for the administration. The resources associated to placebo and Botox treatment were validated by local clinical experts.

## Results

### Cost of illness

**Fig. 2:**
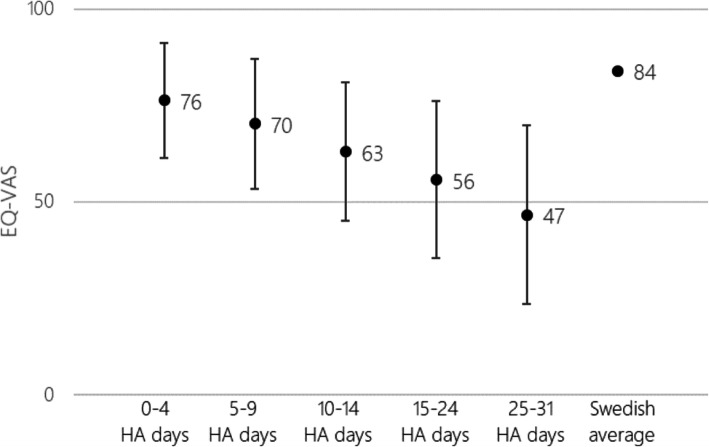
Mean EQ-VAS by number of headache days per month. Key: HA, headache

A total of 454 patients were selected for the COI analysis based on having diagnosed migraine and excluding apparent outliers (*n* = 10). Table 2 presents the patient characteristics stratified by average headache days per month. No substantial differences in age or sex were observed between the headache frequency groups (Kruskal Wallis, age: *p* = 0.0156, sex: *p* = 0.8540). The average age and the gender ratio are representative for the general migraine population, as confirmed by a local clinical expert. It corresponds well with the population modelled for the CE analyses although the COI patient sample was on average somewhat older. The VAS scale ranges from 0 (worst imaginable health state) to 100 (best imaginable health state) [54]. EQ-VAS ranged from 76 in the lowest headache frequency group to 47 in the highest (Fig. 2) (Kruskal Wallis, EQ-VAS: *p* = 0.0001).

**Table 2:** Patient characteristics stratified by headache days per month

Headache days per month	N	Age, mean (SD)	% Female
*0–4 headache days*	99	53.47 (11.53)	90.91
*5–9 headache days*	138	50.40 (11.07)	92.75
*10–14 headache days*	80	51.28 (11.51)	86.25
*15–24 headache days*	74	47.55 (11.27)	87.84
*25–31 headache days*	63	48.90 (13.30)	84.13

***Key***: *SD* Standard Deviation

**Fig. 3:**
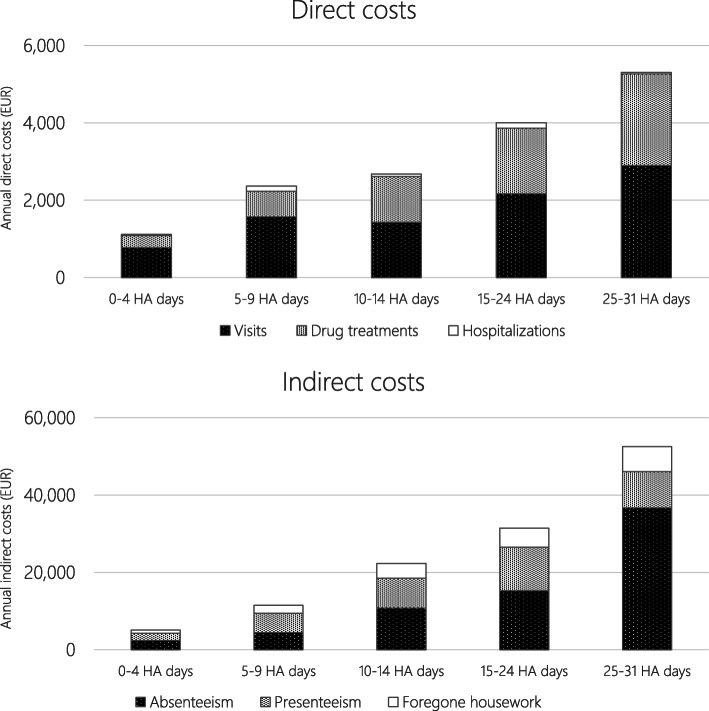
Annual direct and indirect costs by number of headache days. Key: HA, headache

Unsurprisingly, the economic burden in terms of both direct and indirect costs was increasing with number of headache days per month. Average annual headache related direct costs (healthcare utilization in terms of visits and hospitalisations and acute and preventive drug treatment) ranged from EUR 1116 in the group of patients with 0–4 headache days per month, to EUR 5311 in the group with 25–31 headache days (Table 3 and Fig. 3). Healthcare utilization made up the majority of direct costs, ranging from 55% in the 25–31 headache days group to 72% in the 0–4 and 5–9 headache days groups. Health care visits made up the majority of healthcare utilization costs. Drug treatment costs were evenly distributed over acute and preventive treatment costs.

**Table 3:** Annual average direct costs by number of headache days per month

Headache days per month	Direct costs (EUR)		
	Healthcare utilization	Drug treatments	Total
*0–4 headache days*	805	311	1116
*5–9 headache days*	1707	660	2368
*10–14 headache days*	1500	1181	2681
*15–24 headache days*	2312	1697	4009
*25–31 headache days*	2942	2370	5311

Indirect costs were the by far largest cost component (Table 4 and Fig. 3). Average annual indirect costs ranged from EUR 5105 in the 0–4 headache days group to EUR 52,521 in the 25–31 headache days group. Except in the 5–9 headache days group, absenteeism was the largest indirect costs component. In the 5–9 days group, absenteeism made up 39%, presenteeism 43% and foregone housework 18%. In the other groups, absenteeism ranged from 47% to 70%, presenteeism ranged from 18% to 38% and reduced functional ability in terms of forgone housework ranged from 12% to 17%. In the sensitivity analysis using the retail market price approach for foregone household work assuming an hourly cost of EUR 22, the foregone household work cost increased to the following: EUR 1584 in 0–4 headache days, EUR 4183 in 5–9 headache days, EUR 7631 in 10–14 headache days, EUR 10,018 in 15–24 headache days and 12,905 in 25–31 headache days.

**Table 4:** Annual average indirect costs by number of headache days per month

Headache days per month	Indirect costs (EUR)			
	Absenteeism	Presenteeism	Foregone housework	Total
*0–4 headache days*	2381	1934	790	5105
*5–9 headache days*	4445	4985	2085	11,515
*10–14 headache days*	10,808	7676	3805	22,289
*15–24 headache days*	15,312	11,172	4994	31,478
*25–31 headache days*	36,686	9401	6434	52,521

**Fig. 4:**
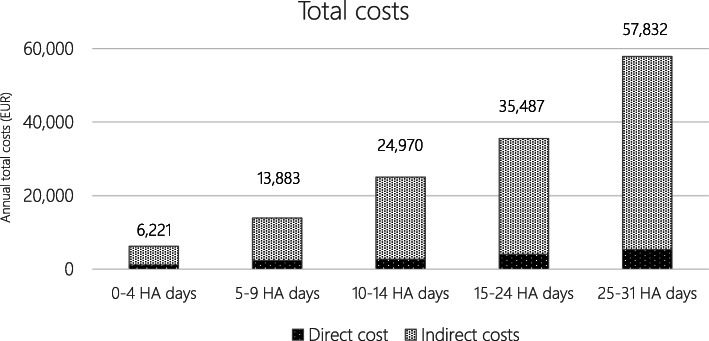
Annual total costs per headache days. Key: HA, headache

Average annual total costs by number of headache days are presented in Fig. 4. Total costs ranged from EUR 6221 in the 0–4 headache days group to EUR 57,832 in the 25–31 headache days group. Indirect costs were the dominating cost component, making up from 82% in the 0–4 headache days group to 91% in the 25–31 headache days group.

Table 5 summarises all COI results which were used to inform the CE analysis, over the modelled headache day intervals.

**Table 5:** Resource use, utilities and work loss per 3-month period by number of headache days per month

Health State	GP/A&E Visits	Avg. number of days absent	Utilities (EQ-VAS)	
			Mean	SD
*0–3 headache days*	0.82	1.44	0.776	0.144
*4–9 headache days*	1.24	3.52	0.702	0.167
*10–14 headache days*	1.34	8.41	0.631	0.180
*15–19 headache days*	1.49	6.81	0.615	0.181
*20–23 headache days*	2.41	19.40	0.474	0.217
*24+ headache days*	2.49	28.22	0.468	0.231

***Key***: *A&E* Accident and emergency; *GP* General practitioner; *HA* Headache

### Cost-effectiveness

#### Base case analysis

The results of the base case analysis are presented in Table 6 for Sweden and in Table 7 for Norway. In Sweden, Botox treatment was associated with 5.711 quality adjusted life years (QALYs) and a cost of EUR 20,700 per patient over a 10-year time horizon. The corresponding for placebo was 5.488 QALYs and a cost of EUR 16,574. Consequently, Botox was associated with 0.223 additional QALYs at an additional cost of EUR 4126 as compared to placebo, resulting in an incremental cost-effectiveness ratio (ICER) of EUR 18,506.

**Table 6:** Base case results, Sweden

	Cost components (EUR)						
	Treatment costs	GP/A&E visits	Hospital	Triptan treatment	Total direct costs		
***Botox***	8535	12,949	1338	472	23,293		
***Placebo***	2749	14,087	1488	522	18,846		
	**Total and incremental results**						
	**Total costs (EUR)**	**Discounted total costs (EUR)**	**Incremental cost (EUR)**	**Total QALYs**	**Discounted QALYs**	**Incremental QALYs**	**ICER**
***Botox***	23,293	20,700	4126	6.505	5.711	0.223	**18,506**
***Placebo***	18,846	16,574		6.257	5.488		

***Key***: *A&E* Accident and emergency; *GP* General practitioner; *ICER* Incremental cost-effectiveness ratio; *QALY* Quality adjusted life years. **Note:** Treatment costs include the administration cost for Botox and the neurologist visits for both Botox and placebo

**Table 7:** Base case results, Norway

	Cost components (EUR)						
	Treatment costs	GP/A&E visits	Hospital	Triptan treatment	Total direct costs		
***Botox***	7751	3177	1549	819	13,297		
***Placebo***	2439	3457	1723	906	8524		
	**Total and incremental results**						
	**Total costs (EUR)**	**Discounted Total costs (EUR)**	**Incremental cost**	**Total QALYs**	**Discounted QALYs**	**Incremental QALYs**	**ICER**
***Botox***	13,297	11,501	4301	6.503	5.480	0.216	**19,954**
***Placebo***	8524	7200		6.255	5.264		

***Key***: *A&E* Accident and emergency; *GP* General practitioner; *ICER* Incremental cost-effectiveness ratio; *QALY* Quality adjusted life years. **Note:** Treatment costs include the administration cost for Botox and the neurologist visits for both Botox and placebo

In Norway, Botox treatment was associated with 5.480 QALYs and a cost of EUR 11,501 per patient over a 10-year time horizon. The corresponding for placebo was 5.264 QALYs and a cost of EUR 7200. Consequently, Botox was associated with 0.216 additional QALYs at an additional cost of EUR 4301 as compared to placebo, resulting in an ICER of EUR 19,954.

Differences in QALYs gained between Sweden and Norway were due to underlying population mortality data (life tables) and different discount rates.

In both Sweden and Norway, willingness to pay (WTP) is related to the severity of the disease. In Sweden, the Dental and Pharmaceutical Benefits Agency (TLV) assessed the severity level of EM as “low to medium” and of CM to “high” [48]. A 2016 analysis of TLV decisions found that the median ICER accepted for non-severe diseases was SEK 280000 (approximately EUR 26000) and for severe diseases (excluding cancer) SEK 363000 (approximately EUR 34000) [55]. In Norway, WTP depend on the absolute shortfall for the disease in question, i.e. the total future health a patient is expected to lose as a result of their condition. NOMA has estimated the absolute shortfall for EM to 5 QALYs and for CM to 11 QALYs, corresponding to WTP levels of NOK 385000 (approximately EUR 35000) for EM and NOK 495000 (approximately EUR 46000) for CM [49].

#### Sensitivity analyses

Analyses of structural changes and additional one-way analyses based on local clinician feedback are presented in Table 8. Results were the most sensitive to the inclusion of indirect costs which is unsurprising considering that it was the by far greatest cost component. When including indirect costs, Botox was dominating in both countries. Following indirect costs, not applying any stopping rule, changing the stopping rule from 30% to 0% and decreasing the time horizon to 5-years had the greatest impact on the results.

**Table 8:** Sensitivity analyses

	Model input		ICER (EUR)	
	Base case	Sensitivity	Sweden	Norway
***Base case***			18,506	19,954
***Analyses of structural changes and altering assumptions***				
Indirect costs	Not included	Included	Dominating	Dominating
Discount rate costs and health effects	3% (Sweden) and 4% (Norway)	0%	17,931	19,250
		5%	18,893	20,132
Stopping rule	30% reduction in 2 cycles	No stopping rule	23,412	24,545
		50% reduction in 2 cycles	17,666	19,163
		0% reduction in 2 cycles	21,825	23,070
Population	All patients	≥1 prior prophylactic	15,160	16,900
		≥3 prior prophylactics	15,764	17,532
Utilities	Estimated from COI database	REPOSE [56]	14,565	15,702
		PREEMPT [18, 24]	15,634	16,804
		IBMS	18,219	19,644
Time horizon	10 years	5 years	21,481	22,645
***Analysis mirroring previous erenumab analysis***				
***Norwegian (NOMA) and Swedish (TLV) erenumab analyses:*** 10-year time horizon^a^, indirect costs not included, ≥3 prior prophylactics^b^, stopping rule 30% reduction in 2 cycles, utilities mapped from MSQ to EQ-5D^c^			16,625	18,462

^a^*NOMA utilised a 10-year time horizon, while the TLV time horizon was confidential. We therefore chose to undertake the analyses using a 10-year time horizon*

^b^*The TLV analyses were undertaken in a population with ≥ 2 prior prophylactics and 30% stopping rules. There is no available data for this combination of population and stopping rule for Botox. The NOMA analysis was however undertaken in a population of ≥ 3 prior prophylactics. We chose to undertake the analyses the ≥ 3 prior prophylactics population, and to use the 30% stopping rule, as NOMA argued for the importance of this specific rule*

^c^*The CGRP analyses utilised utilities mapped from MSQ to EQ-5D, based on clinical trials*

**Fig. 5:**
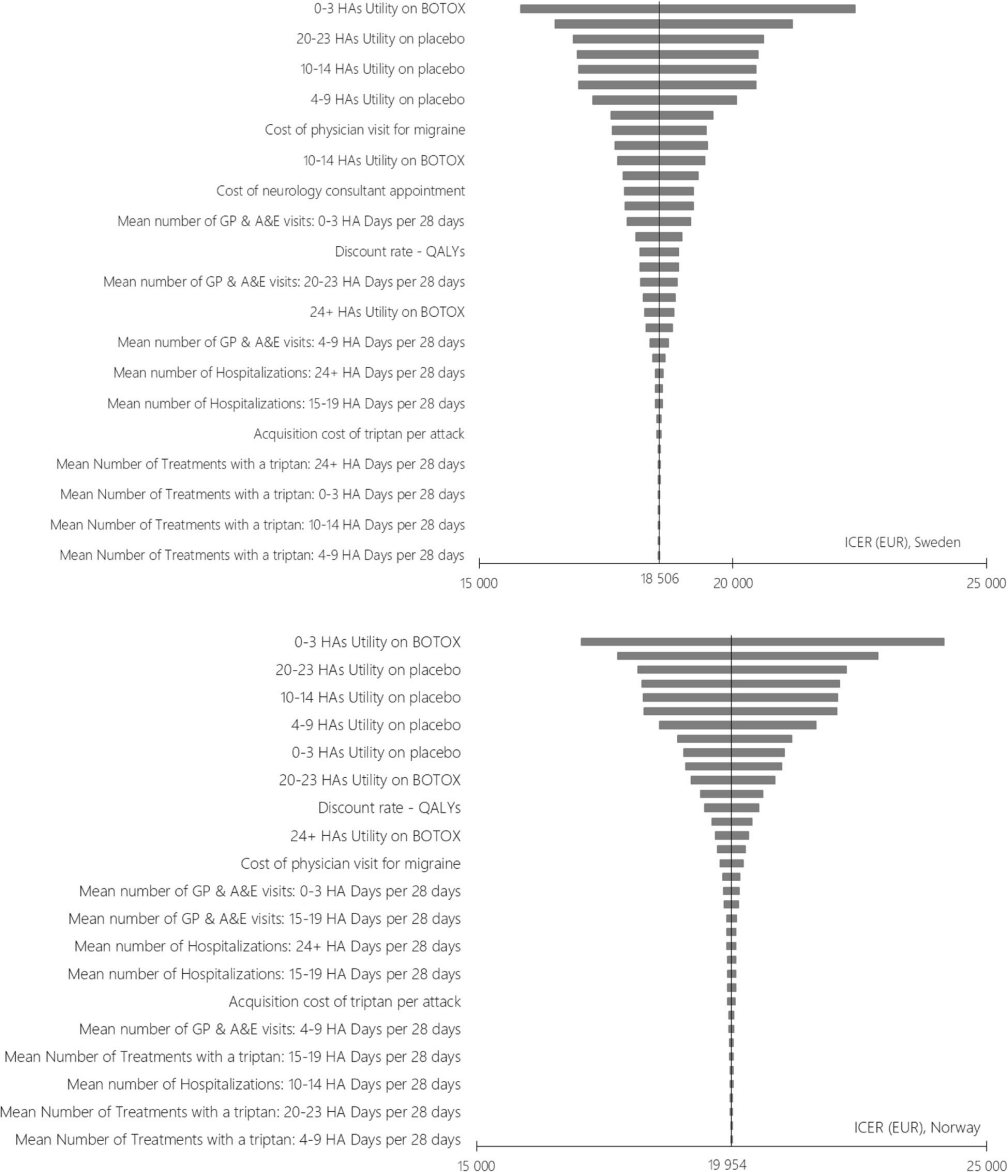
Tornado Diagrams. Key: HA, headache; ICER, incremental cost-effectiveness ratio, QALY, quality adjusted life year

**Fig. 6:**
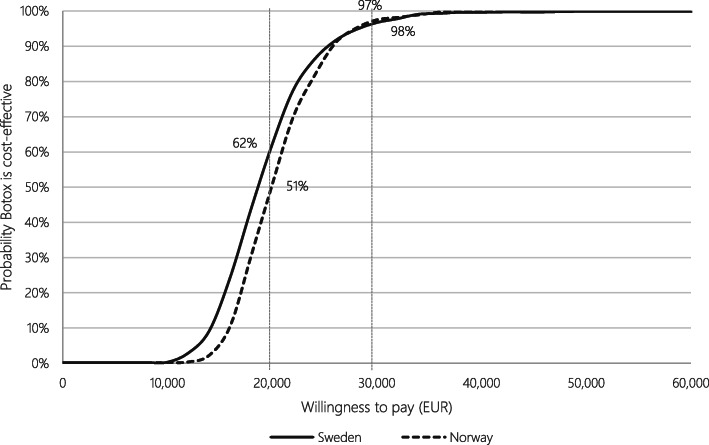
Cost-Effectiveness Acceptability Curve (CEAC). Note: WTP levels of EUR 20,000 and EUR 30,000 are indicated by the grey vertical lines

The Tornado diagrams (Fig. 5) list the one-way sensitivity analyses with the greatest impact on the results in descending order. Variations of utilities had the greatest impact, which is unsurprising considering the QALY gain is completely based on differences in utilities as no difference in survival between the health states was assumed. A probabilistic sensitivity analysis (PSA) was undertaken using 1000 iterations. The outcome showed that results were robust to uncertainty of input parameters. In Sweden, the probabilistic incremental cost was EUR 4081 with corresponding incremental QALYs of 0.220, resulting in a probabilistic ICER of 18,556. In Norway, the probabilistic incremental cost was EUR 4270 with corresponding incremental QALYs of 0.215, resulting in a probabilistic ICER of EUR 19,872. The resulting cost-effectiveness acceptability curves (CEAC) are presented in Fig. 6. At a WTP level of EUR 20,000 there is a 62% probability that Botox is cost-effective in Sweden, and 51% in Norway. At a WTP level of EUR 30,000 there is a 97% and 98% probability that Botox is cost-effective in Sweden and Norway, respectively.

## Discussion

Our COI findings show a clear correlation between headache frequency and increased costs, indicating substantial potential cost savings related to reducing the number of headache days per month. Botox reduces the number of headache days per month in a patient group with few other treatments available. And, as made apparent by the cost-effectiveness analysis, Botox is likely a cost-effective treatment option in Sweden and Norway.

The correlation between productivity losses – both in terms of labour productivity and foregone household work – and headache frequency can be interpreted as a proxy for quality of life. The EQ-VAS results further support this notion and demonstrate that the number of headache days per month is not only a significant cost driver, but also induces a substantial quality of life burden for the sufferers. EQ-VAS in the 25–31 headache days group was almost halved in comparison to the general population, while EQ-VAS in the 0–4 headache days group was almost at general population level. A large majority of the cost burden of migraine was due to indirect costs resulting from reduced productivity and functional ability (ranging from 82% of total costs in the 0–4 headache days group to 91% in the 25–31 headache days group). These results are in line with previous findings [12, 13, 57]. The CE findings indicate that Botox treatment is cost effective in Sweden and Norway, with ICERs well below the previously discussed TLV and NOMA WTP thresholds (ranging from approximately EUR 26,000 to EUR 34,000 in Sweden and from EUR 35,000 to 46,000 in Norway). The results were robust to several sensitivity analyses but were the most sensitive to the inclusion of indirect costs, assumptions on the stopping rule, shortening the time horizon and variations in utilities. The results of this analysis are in line with previous results. A 2013 UK study assessed the CE of Botox in adults in CM compared to placebo, and estimated an ICER of GBP 15,028, assuming a 2-year time horizon and not including indirect costs. When including indirect costs the ICER was reduced to GBP 9422 [24]. An updated analysis resulted in an ICER of GBP 16,306, in CM patients who have previously failed at least three preventive treatments [25]. In 2014, an Italian study comparing Botox to placebo in patients with CM estimated an ICER of EUR 9407, assuming a 2-year time horizon and not including indirect costs. When considering indirect costs, the ICER was reduced to EUR 815 [26]. Likely drivers of differences in these results are utility values used, the country specific Botox price, different assumptions regarding stopping rule, and our relatively higher costs related to productivity losses.

The COI study had several limitations. As inherent with survey studies, there is risk of recall bias. The relatively short time span for recalling headaches and resource use (i.e. 3 months) likely reduced this risk. Additionally, the fact that the 3 months responses were extrapolated to annual costs adds uncertainty as the extrapolation relies on the underlying assumption that migraine severity and intensity is fairly constant over one-year periods. The survey being undertaken in a patient organisation reduces the generalisability of the results, as there is a risk that these patients differ from the general migraine population. Patients may be motivated to participate in such organizations due to large disease burden, resulting in a patient population with heightened severity as compared to general disease populations. Regarding this study, this is likely not a major concern considering the results being stratified over disease severity expressed in monthly average number of headache days. Additionally, several local clinical experts have confirmed that the patient characteristics of our sample corresponds well with the clinical practice migraine patient population.

This study only included patients with diagnosed migraine. Norwegian estimations suggest that approximately 50% of migraine sufferers are undiagnosed [58]. There is however no straightforward way to include these undetected patients. In order to limit the study to actual migraine sufferers, including only diagnosed migraine was the only viable option. The limited patient sample (*N* = 454), and especially when stratified over the headache days subgroups present a statistical challenge made apparent by the relatively high standard deviations obtained for each of the healthcare utilization estimates. Concerns raised regarding the robustness of the results are however reduced by the fact that results are in line with previous literature. A final limitation with the COI study is that results likely underestimated the indirect costs as the survey did not include a question on workforce participation. This renders a risk that patients completely incapacitated by their headache and therefore not in the workforce may have not answered the question related to absenteeism. A proportion of these patients may have answered the questions relating to foregone housework instead, further augmenting the importance of considering unpaid labour and spare time in the analysis. This concern is increased by the fact that our findings regarding productivity losses was lower than previous Swedish results [59], which was also pointed out by clinical experts. It should therefore be noted that the results regarding indirect costs are at risk of being underestimated, and that the total disease costs may be higher than estimated in our study. Additionally, this study utilised conservative approaches in the costing of both presenteeism and foregone household work.

Uncertainty is inherent in all CE modelling. In order to reduce uncertainty related to assumptions and model inputs extensive interviews with local clinical experts were undertaken. Limitations however remained. The CE model was constructed with headache frequency intervals based on the Botox clinical trials, and therefore differed slightly from the approach used in the COI estimations. This was solved by estimating the model inputs sourced from the COI study using the model intervals. The comparator, placebo, was selected in absence of an established standard of care, and to be in line with the Botox clinical trials. The choice was validated by local clinical experts. The PREEMPT placebo patients received saline injections and were permitted to remain on their standard acute headache treatment. The trials observed considerable headache frequency reductions in the placebo group [18–21] which are not likely to be observed in a real-world setting, making the choice of comparator conservative. No CE analyses comparing Botox to CGRP inhibitors (erenumab and fremanezumab) were undertaken due to lack of reliable comparative efficacy data and due to lack of price information: erenumab and fremanezumab have been launched in the Nordics and are reimbursed in accordance with confidential price agreements. However, as suggested by TLV, an indirect comparison could be done by comparing the ICERs from the analyses comparing each active substance to placebo (given similar QALY differences) [48]. For Sweden, TLV reports the difference in QALYs gained to be 0.20 and 0.21 for fremanezumab and erenumab, respectively, vs placebo, resulting in ICERs of SEK 505,000 (EUR 47,690) and SEK 557,000 (EUR 52,601), respectively [48]. Both analyses used confidential net prices. For Norway, NoMA reports the difference in QALYs to be 0.17 for erenumab vs placebo (ICER not reported as the analysis was based on a confidential net price) [49]. In the present study, when attempting to mirror the CGRP analyses as closely as possible (patients with ≥3 previous treatments, 10-year time horizon, 30% stopping rule and using utility values mapped from MSQ to EQ-5D) the difference in QALYs for Botox vs placebo were 0.209 and with a corresponding ICER of EUR 16,625 in Sweden and 0.203 QALY difference with a corresponding ICER of 18,462 in Norway.

## Conclusions

In conclusion, in people with migraine the number of average monthly headache days is clearly related to lower QoL and higher costs, indicating considerable potential costs-savings in reducing the number of headache days. The study also shows that the main cost driver for migraine are productivity losses. The CE results indicate that considering a 10-year time horizon, Botox is a cost-effective treatment option for migraine in Sweden and Norway. When considering indirect costs, Botox is dominating, i.e. Botox provide additional outcome at a lower cost.

## Data Availability

The datasets generated and/or analysed during the current study are not publicly available due to legal boundaries related to the sharing of individual data.

## Abbreviations

ASA: Acetylsalicylic acid
CE: Cost-Effectiveness
CGRP: Calcitonin gene-related peptide
CM: Chronic migraine
COI: Cost of illness
COMPEL: The chronic migraine onabotulinumtoxina prolonged efficacy open label
CPI: Consumer price index
ECB: European central bank
EQ-VAS: EuroQol visual analogue scale
EU: European union
HA: Headache
ICER: Incremental cost-effectiveness ratio
MSQ: Migraine specific questionnare
NOK: Norwegian krone
NOMA: Norwegian medicines agency
NSAID: Nonsteroidal anti-inflammatory drugs
PREEMPT: Phase 3 research evaluating migraine prophylaxis therapy
PSP: Pharmacy selling price
QALY: Quality adjusted life year
QoL: Quality of life
SEK: Swedish krona
SPC: Summary of product characteristics
TLV: The dental and pharmaceutical benefits agency *[Tandvårds och Läkemedelsförmånsnämnden]*
UK: United Kingdom
VAT: Value added tax
WTP: Willingness to pay

## References

[1] Dahlöf CH, JE. Migrän 2019. Available from: https://www.internetmedicin.se/page.aspx?id=251

[2] Linde M, Dahlöf C (2004). Attitudes and burden of disease among self-considered migraineurs—a nation-wide population-based survey in Sweden. Cephalalgia.

[3] Norwegian health informatics (NHI). Migraines, overview. 2018. Available from: https://nhi.no/sykdommer/hjernenervesystem/hodepiner/migrene-oversikt/.

[4] Burton WN, Conti DJ, Chen C-Y, Schultz AB, Edington DW (2002). The economic burden of lost productivity due to migraine headache: a specific worksite analysis. J Occup Environ Med.

[5] Terwindt G, Ferrari M, Tijhuis M, Groenen S, Picavet H, Launer L (2000). The impact of migraine on quality of life in the general population: the GEM study. Neurology.

[6] Burton WN, Landy SH, Downs KE, Runken MC (2009). The impact of migraine and the effect of migraine treatment on workplace productivity in the United States and suggestions for future research. Mayo Clin Proc.

[7] Molarius A, Tegelberg Å, Öhrvik J (2008). Socio-economic factors, lifestyle, and headache disorders—a population-based study in Sweden. Headache.

[8] Dueland AN, Leira R, Burke TA, Hillyer EV, Bolge S (2004). The impact of migraine on work, family, and leisure among young women–a multinational study. Curr Med Res Opin.

[9] Vo P, Fang J, Bilitou A, Laflamme AK, Gupta S (2018). Patients’ perspective on the burden of migraine in Europe: a cross-sectional analysis of survey data in France, Germany, Italy, Spain, and the United Kingdom. J Headache Pain.

[10] Xu R, Insinga RP, Golden W, Hu XH (2011). EuroQol (EQ-5D) health utility scores for patients with migraine. Qual Life Res.

[11] Negro A, Sciattella P, Rossi D, Guglielmetti M, Martelletti P, Mennini FS (2019). Cost of chronic and episodic migraine patients in continuous treatment for two years in a tertiary level headache Centre. J Headache Pain.

[12] Linde M, Gustavsson A, Stovner LJ, Steiner TJ, Barré J, Katsarava Z (2012). The cost of headache disorders in Europe: the Eurolight project. Eur J Neurol.

[13] Hjalte F, Olofsson S, Persson U, Linde M (2019). Burden and costs of migraine in a Swedish defined patient population–a questionnaire-based study. J Headache Pain.

[14] NEL Nevrologi. Retningslinjer for Botox-behandling ved kronisk migrene 2018. Available from: https://nevrologi.legehandboka.no/handboken/diverse/retningslinjerveiledere/retningslinjer-for-botox-behandling-ved-kronisk-migrene/

[15] Norsk legemiddelhåndbok. T6.2.1 Migrene 2018. Available from: https://www.legemiddelhandboka.no/T6.2.1

[16] European Medicines Agency (EMA). Summary of Product Characteristics. BOTOX® 50 Allergan Units, Powder for Solution for Injection. European Medicines Agency (EMA). 2019

[17] Headache Classification Committee of the International Headache Society (2013). The international classification of headache disorders, (beta version). Cephalalgia.

[18] Aurora SK, Winner P, Freeman MC, Spierings EL, Heiring JO, DeGryse RE (2011). OnabotulinumtoxinA for treatment of chronic migraine: pooled analyses of the 56-week PREEMPT clinical program. Headache J Head Face Pain.

[19] Dodick DW, Turkel CC, DeGryse RE, Aurora SK, Silberstein SD, Lipton RB (2010). OnabotulinumtoxinA for treatment of chronic migraine: pooled results from the double-blind, randomized, placebo-controlled phases of the PREEMPT clinical program. Headache J Head Face Pain.

[20] Aurora S, Dodick DW, Turkel C, DeGryse R, Silberstein S, Lipton RB (2010). OnabotulinumtoxinA for treatment of chronic migraine: results from the double-blind, randomized, placebo-controlled phase of the PREEMPT 1 trial. Cephalalgia.

[21] Diener H, Dodick DW, Aurora S, Turkel C, DeGryse R, Lipton RB (2010). OnabotulinumtoxinA for treatment of chronic migraine: results from the double-blind, randomized, placebo-controlled phase of the PREEMPT 2 trial. Cephalalgia.

[22] Blumenfeld AM, Stark RJ, Freeman MC, Orejudos A, Adams AM (2018). Long-term study of the efficacy and safety of OnabotulinumtoxinA for the prevention of chronic migraine: COMPEL study. J Headache Pain.

[23] Davies B, Gaul C, Martelletti P, García-Moncó JC, Brown S (2017). Real-life use of onabotulinumtoxinA for symptom relief in patients with chronic migraine: REPOSE study methodology and baseline data. J Headache Pain.

[24] Batty AJ, Hansen RN, Bloudek LM, Varon SF, Hayward EJ, Pennington BW (2013). The cost-effectiveness of onabotulinumtoxinA for the prophylaxis of headache in adults with chronic migraine in the UK. J Med Econ.

[25] Hollier-Hann G, Curry A, Onishchenko K, Akehurst R, Ahmed F, Davies B (2020). Updated cost-effectiveness analysis of onabotulinumtoxinA for the prevention of headache in adults with chronic migraine who have previously received three or more preventive treatments in the UK. J Med Econ.

[26] Ruggeri M (2014). The cost effectiveness of Botox in Italian patients with chronic migraine. Neurol Sci.

[27] Huvudvärksförbundet ("the headache association"). 2020 [Available from: http://www.huvudvarksforbundet.se/

[28] Stewart WF, Lipton RB, Whyte J, Dowson A, Kolodner K, Liberman J (1999). An international study to assess reliability of the Migraine Disability Assessment (MIDAS) score. Neurology.

[29] FASS. Botox Allergan 2020 [Available from: https://www.fass.se/LIF/product?docType=30&specId&userType&nplId=20091117000049&scrollPosition=400

[30] Felleskatalogen. Botox Allergan 2019 [Available from: https://www.felleskatalogen.no/medisin/botox-allergan-547057

[31] Södra sjukvårdsregionen. Regionala priser och ersättningar för södra sjukvårdsregionen 2019 [Available from: https://sodrasjukvardsregionen.se/verksamhet/avtal-priser/regionala-priser-och-ersattningar-foregaende-ar/

[32] Helsedirektoratet. Regelverk ISF 2018, DRG-liste somatikk 2018 [Available from: sieringsordninger/innsatsstyrt-finansiering-isf-og-drg-systemet/innsatsstyrt-finansiering-isf#regelverk-isf-2018

[33] Statistics Sweden. Genomsnittlig månadslön efter yrke 2018 [Available from: https://www.scb.se/hitta-statistik/statistik-efter-amne/arbetsmarknad/loner-och-arbetskostnader/lonestrukturstatistik-landstingskommunal-sektor/pong/tabell-och-diagram/genomsnittlig-manadslon-efter-yrke/

[34] Legemiddelverket. Enhetskostnadsdatabase 2018 [Available from: https://legemiddelverket.no/Documents/Offentlig%20finansiering%20og%20pris/Dokumentasjon%20til%20metodevurdering/Enhetskostnadsdatabase_kostnader_0040618.pdf

[35] Norsk Legeforening. Normaltariff for avtalespesialister 2018–2019 [Available from: https://normaltariffen.legeforeningen.no/pdf/Normaltariff_2018.pdf

[36] Norsk legemiddelhåndbok. L6.2.1 Selektive 5-HT1-reseptoragonister 2017 [Available from: https://www.legemiddelhandboka.no/L6.2.1/Legemidler_ved_nevrologiske_sykdommer#Lk-06-nevrol-1457

[37] Statistics Sweden. Medellöner i Sverige 2018 [Available from: https://www.scb.se/hitta-statistik/sverige-i-siffror/utbildning-jobb-och-pengar/medelloner-i-sverige/

[38] OECD. Hours worked - Total, Hours/worker, 2018 or latest available 2018 [Available from: https://data.oecd.org/emp/hours-worked.htm

[39] Transportstyrelsen. Konsekvensutredning - Ändringsföreskrift trafikregler för luftfart 2015 [2020-02-17]. Available from: https://transportstyrelsen.se/globalassets/global/regler/remisser/luftfart/konsekvensutredning-150611.pdf

[40] Hemfrid. Weekly cleaning and house chores 2020 [Available from: https://www.hemfrid.se/?gclid=CjwKCAiA1rPyBRAREiwA1UIy8CeBqKyHX2ToIiM68FaRox2-vAS6D15Taoct5sb0EMcXt8zg3DPtNhoCx-MQAvD_BwE

[41] HomeMaid. Weekly cleaning and house chores 2020 [Available from: https://homemaid.se/vara-tjanster/hemstadning/

[42] Statistics Sweden. Konsumentprisindex (1980=100), fastställda tal [Consumer Price Index] 2020 [Available from: https://www.scb.se/hitta-statistik/statistik-efter-amne/priser-och-konsumtion/konsumentprisindex/konsumentprisindex-kpi/pong/tabell-och-diagram/konsumentprisindex-kpi/kpi-faststallda-tal-1980100/

[43] Statistics Norway. Consumer price index 2020 [Available from: https://www.ssb.no/en/priser-og-prisindekser/statistikker/kpi/maaned

[44] European Central Bank (ECB). Foreign Exchange reference rates - Swedish Krona (SEK), 2019 average 2019 [Available from: https://www.ecb.europa.eu/stats/policy_and_exchange_rates/euro_reference_exchange_rates/html/eurofxref-graph-sek.en.html

[45] European Central Bank (ECB). Foreign Exchange reference rates - Norwegian Krone (NOK), 2019 average 2019 [Available from: https://www.ecb.europa.eu/stats/policy_and_exchange_rates/euro_reference_exchange_rates/html/eurofxref-graph-nok.en.html

[46] Statistics Sweden. Tidsanvändningsundersökningen (2010) - Hemarbete mer jämlikt (The Swedish Time Use Survey (2010) - Men increase their share of unpaid housework) 2011 [Available from: https://www.scb.se/en/finding-statistics/statistics-by-subject-area/living-conditions/living-conditions/the-swedish-time-use-survey/pong/statistical-news/the-swedish-time-use-survey-2010/

[47] Verbooy K, Hoefman R, Van Exel J, Brouwer W (2018). Time is money: investigating the value of leisure time and unpaid work. Value Health J.

[48] The Dental and Pharmaceutical Benefits Agency (TLV). Underlag för beslut om subvention - Nyansökan Nämnden för läkemedelsförmåner Ajovy (fremanezumab) Utvärderad indikation 2019 [Available from: https://www.tlv.se/download/18.27af28ab16e5ee38db11d297/1573647481854/bes191024_underlag_ajovy.pdf

[49] The Norwegian Medicines Agency (NOMA). Hurtig metodevurdering ved forhåndsgodkjent refusjon §2 Aimovig (erenumab) til profylaktisk behandling av migrene. 2019 [Available from: https://legemiddelverket.no/Documents/Offentlig%20finansiering%20og%20pris/Metodevurderinger/A/Aimovig_migrene_2019.pdf

[50] The Dental and Pharmaceutical Benefits Agency (TLV). Ändring i Tandvårds- och läkemedelsförmånsverkets allmänna råd (TLVAR 2003:2) om ekonomiska utvärderingar 2017 [Available from: https://www.tlv.se/download/18.467926b615d084471ac3230c/1510316374332/TLVAR_2017_1.pdf

[51] The Dental and Pharmaceutical Benefits Agency (TLV). Hälsoekonomi (in english: health economics) 2020 [Available from: https://www.tlv.se/lakemedel/halsoekonomi.html

[52] Gillard PJ, Devine B, Varon SF, Liu L, Sullivan SD (2012). Mapping from disease-specific measures to health-state utility values in individuals with migraine. Value Health J.

[53] Blumenfeld AM, Varon SF, Wilcox TK, Buse DC, Kawata AK, Manack A (2011). Disability, HRQoL and resource use among chronic and episodic migraineurs: results from the international burden of migraine study (IBMS). Cephalalgia.

[54] Feng Y, Parkin D, Devlin NJ (2014). Assessing the performance of the EQ-VAS in the NHS PROMs programme. Qual Life Res.

[55] Svensson M, Nilsson F (2016) TLV: s betalningsvilja för nya läkemedel har analyserats: Kostnadseffektivitet och sjukdomens svårighetsgrad avgörande för subvention-Cancerläkemedel får kosta mer. Läkartidningen 113(28–30). https://lakartidningen.se/halsoekonomi/2016/07/tlvs-betalningsvilja-for-nya-lakemedel-haranalyserats/. Accessed 20 July 2020. 27404777

[56] Ahmed F, Gaul C, Garcia-Monco JC, Sommer K, Martelletti P (2019). An open-label prospective study of the real-life use of onabotulinumtoxinA for the treatment of chronic migraine: the REPOSE study. J Headache Pain.

[57] Bloudek L, Stokes M, Buse D, Wilcox T, Lipton RB, Goadsby P (2012). Cost of healthcare for patients with migraine in five European countries: results from the international burden of migraine study (IBMS). J Headache Pain.

[58] Headaches Norway. Migraine. Available from: https://hodepinenorge.no/hodepine/migrene/. Accessed 20 July 2020.

[59] Hjalte F, Olofsson S, Persson U. Sjukdomsbördan vid migrän i Sverige - en enkätstudie av resurskonsumption och livskvalitet (eng: The burden of illness og migraine in Sweden - a questionnaire study of resource utilization and quality of life) - IHE Rapport 2018:2. The Swedish Institute for Health Economics (IHE). 2018(2018:4):82.

